# Improvement of Catalyst Layers in Direct Methanol Fuel Cells Using Dual-Electrode Carbon Nanotube Carrier Structure

**DOI:** 10.3390/nano16070430

**Published:** 2026-03-31

**Authors:** Bo Yang, Xuejiao Li, Dacheng Zhang, Zhengang Zhao

**Affiliations:** 1 Faculty of Information Engineering and Automation, Kunming University of Science and Technology, Kunming 650500, China; bo.yang@kust.edu.cn (B.Y.); lixuejiao@stu.kust.edu.cn (X.L.); zhengang.zhao@kust.edu.cn (Z.Z.); 2 Yunnan Key Lab of Computer Technology Applications, Kunming 650500, China; 3 Yunnan Key Lab of Green Energy, Electric Power Measurement Digitalization, Control and Protection, Kunming 650500, China

**Keywords:** direct methanol fuel cell, carbon nanotubes, dual-electrode catalyst support, mass transport enhancement, interfacial resistance reduction

## Abstract

The sole use of carbon nanotubes (CNTs) as single-electrode carriers in direct methanol fuel cells (DMFCs) creates structural disparities that increase resistance, impair catalyst utilization, and limit discharge duration. This study presents a novel dual-electrode CNT-based carrier structure designed to enhance mass transport and electron conduction, thereby improving DMFC power output and durability. The CNTs were grown in situ via nitrogen sintering onto the microporous layer, with parameters optimized to enhance surface morphology and conductivity. The impact of this dual-electrode CNT carrier was evaluated through microstructural characterization, cyclic voltammetry, electrochemical performance testing, and service life assessment. Results demonstrate that the structure improves catalyst dispersion, electrochemical active surface area (ECSA), and charge transfer efficiency, while reducing ohmic resistance and charge transfer impedance. Compared to traditional carbon black (CB) carriers, peak power increased by 51.06%. Under China Light Vehicle Test Cycle (CLTC) conditions, discharge duration increased by a factor of 1.7, indicating higher energy efficiency. These improvements are attributed to the dual-electrode architecture’s synergistic enhancement of proton transport, balanced electrochemical kinetics, and reduced interfacial resistance.

## 1. Introduction

Direct methanol fuel cells (DMFCs) have attracted significant research attention in the energy sector thanks to their high energy density, convenient storage and portability, and low environmental impact [1]. However, traditional catalyst layers that use carbon black (CB) as a carrier suffer from particle agglomeration and detachment during electrochemical reactions, reducing the availability of active sites, increasing mass transfer resistance, and ultimately leading to low catalyst utilization and shortened DMFC lifespan [2,3].

Researchers are continually seeking methods to enhance catalyst-carrier interactions and improve particle anchoring. An ideal carbon support must meet two key conditions [4]: (1) a high specific surface area to facilitate the dispersion of precious metal catalysts and expose more active sites, thereby enhancing methanol oxidation rates; and (2) high graphitization for excellent electronic conductivity to reduce internal cell resistance and improve energy conversion efficiency. Consequently, optimizing the catalyst layer structure to reduce multiphase transport resistance (gas, water, heat, and electricity) and increase the electrochemical reaction area is key to enhancing DMFC performance.

The support material critically influences catalyst dispersion, stability, anchoring performance, and atomic utilization efficiency, thereby determining overall catalytic performance. Recent studies prioritize novel supports with enhanced conductivity and high specific surface area to optimize these characteristics. In particular, recent advances have demonstrated that precisely tuning the support defect chemistry and constructing 3D interconnected carbon nanonetworks can drastically boost the mass-specific activity and operational durability of DMFCs [5,6]. Building on this, emerging research further highlights the critical role of support-induced electronic effects and heterostructure engineering in accelerating the sluggish methanol oxidation reaction (MOR) kinetics while effectively mitigating CO poisoning [7,8]. In this context, carbon nanotubes (CNTs) have emerged as a prominent catalyst carrier due to their ideal properties. Using CNTs as catalyst carriers for both the anode and cathode has the potential to optimize mass transport and electron conduction, thereby improving DMFC power density and durability and opening new possibilities for portable power sources and micro-devices [9].

As summarized in Table 1, various Pt-based catalysts and support modifications have been explored to enhance DMFC performance. Notable examples include the work by Chang et al. [10], who used ozone heat treatment on carbon black to introduce oxygen-containing groups for better Pt dispersion and CO tolerance. Other strategies include: controlling morphology with core-shell structures like Au–Pt/Au–Pd [11]; leveraging strong metal-support interactions via in situ-synthesized PtPd nanocubes [12]; and employing composite supports such as TiO_2_-graphene aerogels [13] or silane-modified rGO [14] to boost mass activity and current density. However, while modified traditional carbon supports offer adequate initial activity, their long-term stability is fundamentally limited by carbon corrosion and catalyst agglomeration. To overcome this, the dual-electrode CNT carrier developed here provides a highly graphitized 3D interconnected network. This structure not only enhances mass transport beyond conventional supports (Table 1) but also secures Pt nanoparticles, thereby improving durability.

While studies on modified carbon blacks, TiO_2_-GA, and NH_2_-rGO demonstrate progress through surface functionalization and morphology control, their performance is ultimately constrained by the intrinsic properties of the base support. To overcome these limitations, research has evolved toward engineered one-dimensional nanostructures, specifically carbon nanofibers (CNFs) and CNTs. These materials offer superior and tailorable characteristics, including high aspect ratio, tunable porosity, exceptional axial conductivity, and robust mechanical strength. This combination of properties provides a more advantageous platform for catalyst dispersion, electron conduction, and mass transport.

As summarized in the literature, carbon nanofibers [15,16] and CNTs [17,18,19] are leading candidates. For instance, nitrogen-functionalized multi-walled CNTs have been shown to enhance Pt deposition and activity [20], while helical carbon nanofiber structures improved charge transfer in battery anodes [21]. Similar advances in dispersion, active-site preservation, and stability have been reported for various nanofiber and nanotube architectures [22,23,24,25].

While existing studies confirm the efficacy of CNTs as carriers, most have focused on single-electrode optimization, applying CNTs independently to either the anode or the cathode. When employed solely as single-electrode carriers in DMFCs, CNTs can increase interfacial resistance and imbalance reaction kinetics due to structural disparities. This impairs catalyst dispersion and atom utilization efficiency, limiting discharge duration. This approach overlooks the coupling required between the dual electrodes in a complete electrochemical system. The use of mismatched support materials can trigger systemic issues that limit cell performance.

1. Single-electrode optimization may exacerbate disparities between the anode and cathode in terms of microstructure, conductivity, and hydrophobicity/hydrophilicity, leading to significant contact resistance at the electrode/membrane interfaces.
2. The mismatch between the kinetics of the methanol oxidation reaction (MOR) at the anode and the oxygen reduction reaction (ORR) at the cathode limits the overall cell’s current output and voltage stability.
3. Asymmetric electrode structures can hinder the coordinated transport of reactants (methanol, oxygen) and products (carbon dioxide, water) across both electrodes, exacerbating methanol crossover and reducing water management efficiency.
4. The aforementioned issues collectively result in limited peak power density enhancement, low energy conversion efficiency, short discharge duration under dynamic operating conditions, and rapid performance decay.

Therefore, the conventional single-electrode improvement strategy is insufficient. To holistically overcome these interconnected problems, a paradigm shift towards a dual-electrode, synergistically designed CNT support structure is crucial. Such an architecture, serving both the anode and the cathode in concert, is the key to simultaneously reducing interfacial resistance, balancing reaction kinetics, enhancing multiphase transport, and thereby improving both the power output and the long-term durability of DMFCs. To address this need, the core objective of this study is to construct and evaluate a novel dual-electrode CNT-based catalyst support structure.

The rest of the paper is organized as follows. Section 2 details the methodology, including the design rationale for the dual-electrode CNT carrier and the approach taken for methanol transport optimization. Section 3 describes the experimental procedures, encompassing CNT pretreatment, slurry preparation, membrane electrode assembly, and the testing setup. Section 4 presents and discusses the results from microstructural characterisation, electrochemical analysis (cyclic voltammetry, impedance), power output evaluation, and discharge performance assessment. Finally, Section 5 summarizes the key findings, states the conclusions drawn from this work, and suggests potential directions for future research.

## 2. Methodology

The catalyst layer, where electrochemical reactions occur in DMFCs, exhibits the most complex internal structure and physical processes within the cell. Composed primarily of catalyst particles, a support material, and a perfluorosulfonic acid ionomer (Nafion^®^), this layer’s performance relies critically on the catalyst support. As a key structural component, the support facilitates reactant mass transfer from the flow fields while providing additional active sites that enhance electrocatalytic efficiency when appropriately modified. In this work, multi-walled carbon nanotubes (MWCNTs) are employed to construct a 3D conductive network, which significantly improves the triple-phase boundary (TPB) density. MWCNTs are chosen due to their excellent chemical inertness, high oxidative stability, outstanding electronic conductivity, and large specific surface area. These characteristics establish them as ideal heterogeneous catalyst supports for electrochemical applications. A key parameter for measuring catalyst utilization is the electrochemical active surface area (ECSA). Modifying the catalyst support can improve the dispersion of Pt nanoparticles and thus increase the ECSA. This enhancement is attributed to the high aspect ratio of CNTs, which prevents Pt agglomeration. The ECSA can be calculated using cyclic voltammetry (CV):

(1) $$\mathrm{ECSA}=\frac{Q_{H}}{m_{\mathrm{Pt}}\times q_{H}}$$

where $Q_{H}$ is the hydrogen adsorption charge; $m_{\mathrm{Pt}}$ is the loading of Pt catalyst; and $q_{H}$ is the charge required for monolayer hydrogen adsorption.

### 2.1. Methanol Transport Optimization

The performance of DMFCs during operation is governed by a complex interplay of reaction kinetics and mass transfer limitations. Methanol transport through porous electrodes relies on convective flow (Darcy’s law) and diffusion (Fick’s law). Concurrently, the conductivity of the CNT network follows percolation theory. Detailed theoretical formulations of these underlying physical principles are provided in the Appendix A. In the proposed design, the integrated CNT framework addresses both kinetic and mass-transfer challenges: it increases the exchange current density by reducing the activation energy for the methanol oxidation reaction (MOR), while its hierarchical pore structure enhances the effective diffusion coefficient by reducing tortuosity.

To evaluate the internal resistance and degradation mechanisms, electrochemical impedance spectroscopy (EIS) is employed [26] with an equivalent circuit model (ECM). The physical significance of internal resistance ($R_{\mathrm{int}}=\sum_{i=1}^{5}R_{i}$) includes: the bulk ohmic resistance of the electrolyte and membrane; the contact resistance between the catalyst layer and the current collector; the resistance caused by CO-like byproduct adsorption; the mass transfer resistance of methanol within the porous network; and the charge transfer resistance at the anode/cathode interfaces. This multi-component model is necessary to decouple the overlapping time constants of the complex triple-phase boundary reactions in DMFCs. The overall cell voltage and power output are determined by:

(2) $$U=E_{\mathrm{OCV}}-\eta -IR_{\mathrm{int}},\;P=UI$$

where η is the overpotential. Polarization curves and power density plots can be used to evaluate the impact of CNT loading on the net power gain. Reducing $R_{\mathrm{int}}$ through CNT integration directly enhances *U* and *P*.

### 2.2. CNT-DMFC Structural Design and Synthesis

**Figure 1:**
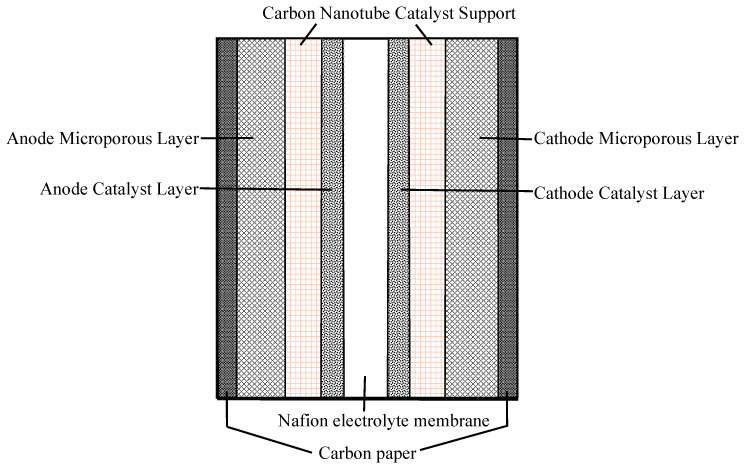
Schematic of the CNT-DMFC MEA structure.

Toray carbon paper was employed as the gas diffusion layer (GDL) support due to its high porosity and mechanical stability. To further suppress methanol crossover, a gradient MPL design was implemented, incorporating CNTs to refine the pore size distribution. The microporous layer (MPL) was fabricated by brushing a homogeneous mixture of carbon powder and polytetrafluoroethylene (PTFE) onto the GDL. The hydrophobic/hydrophilic balance is tuned by the CNT/PTFE ratio, ensuring efficient ${\mathrm{CO}}_{2}$ removal at the anode. The structure design is shown in Figure 1.

This design offers three primary functional advantages:

1. CNTs as cathode supports increase water transport resistance within the catalyst layer, promoting back-diffusion to the anode through capillary-driven flow in hierarchical pores. This effectively mitigates the cathode flooding common in high-current density operations.
2. The hierarchical pore structure of CNTs mitigates methanol crossover through size-exclusion effects and nanochannel selectivity, effectively blocking methanol molecules while facilitating CO_2_ release. This reduces mass transfer limitations and anode poisoning.
3. The structural interconnectivity of the CNT network provides a robust framework that resists catalyst detachment during long-term cycling. This architecture further enhances mechanical strength through efficient stress distribution, thereby maintaining catalyst stability.

## 3. Experiment

Building on the foundational processes of CNT pretreatment and slurry formulation, MEA fabrication is the pivotal phase in constructing a high-performance DMFC. This section delineates the integrated manufacturing protocol, encompassing proton exchange membrane activation, microporous layer optimization, and in situ CNT growth via nitrogen sintering. The objective is to engineer a dual-electrode catalyst support structure that mitigates interfacial resistance and enhances mass transport kinetics. This methodology bridges material synthesis with structural engineering, culminating in an MEA with hierarchical porosity and balanced electrode kinetics. Subsequent validation through a MEMS-based single-cell configuration and a multi-parameter testing platform quantifies the efficacy of this design in elevating power density and operational durability.

### 3.1. CNTs Pretreatment

CNTs inherently contain surface impurities, including incompletely graphitized carbonaceous residues and residual metal catalysts (e.g., Ni, Fe) from chemical vapor deposition (CVD) synthesis. Pretreatment is essential to enhance catalyst adhesion and stability. The process typically involves reflux in strong oxidizing agents to remove impurities and generate oxygen-containing functional groups (e.g., carboxyl and hydroxyl groups). Although nanotube shortening may reduce the specific surface area, these functional groups enhance hydrophilicity. They facilitate methanol/water adsorption through hydrogen bonding, thereby improving mass-transport kinetics at the DMFC anode.

In this study, CNTs were pretreated with concentrated nitric acid of 68 wt% following the procedure:

1. Disperse 500 mg of CNTs (diameter: 3∼15 nm) in 500 mL of concentrated HNO_3_ solution.
2. Sonicate the mixture at 40 kHz for 30 min to disaggregate CNT bundles.
3. Stir magnetically at 300 rpm for 24 h at 25 °C to balance impurity removal with structural preservation.
4. Wash repeatedly with deionized water until the filtrate reaches pH-neutrality, then centrifuge at 10,000 rpm for 5 min to remove acid-etched debris.
5. Dry the purified CNTs under vacuum at 60 °C for three hours to preserve the surface functional groups.

The necessity and efficacy of our rigorous CNT pre-treatment and high-temperature nitrogen sintering protocols are strongly supported by recent literature. As corroborated by recent studies [27,28], pristine CNTs intrinsically suffer from severe agglomeration due to strong van der Waals forces; therefore, extensive surface engineering and dispersion treatments are mandatory to achieve a homogeneous catalytic network. Furthermore, while conventional chemical oxidations often disrupt the carbon lattice, high-temperature thermal treatments in inert atmospheres (such as our $N_{2}$ sintering) have been proven to effectively eliminate amorphous carbon defects and significantly restore the high crystallinity of the CNTs [28,29]. This highly crystalline, defect-minimized, and uniformly dispersed CNT architecture ensures robust π-electron conjugation, thereby maximizing electronic conductivity and interfacial durability in harsh fuel cell operations, which perfectly explains the superior electrochemical stability observed in our CNT-DMFC system [30]. It should be noted that to ensure environmental sustainability and safety, the highly acidic filtrate generated from this process was strictly neutralized with an alkaline solution and subsequently collected by a certified hazardous waste facility.

### 3.2. CNT Slurry Preparation

The CNT slurry was formulated using multi-walled carbon nanotubes (MWCNTs), polyvinyl alcohol (PVA) as a dispersant, and Triton X-100 as a surfactant. This protocol leverages synergistic dispersion mechanisms: (1) PVA polymer chains adsorb onto CNT surfaces, creating steric hindrance; (2) Triton X-100 imparts negative surface charges via its hydrophilic head groups, preventing re-agglomeration through electrostatic repulsion. The optimized preparation steps are as follows.

1. Place 200 mg of acid-pretreated CNTs (diameter: 3∼15 nm) in a beaker.
2. Add 6 mL of deionized water and 100 mg of PVA (1∼5 wt%).
3. Stir magnetically at 500 rpm for 10 min for preliminary mixing.
4. Sonicate the mixture for 24 h to debundle CNT aggregates.
5. Add 100 mg of Triton X-100 and 0.3 mL of N-methyl-pyrrolidone (NMP).
6. Sonicate again to ensure uniform dispersion.
7. Centrifuge at 10,000 rpm for 5 min to remove any remaining undispersed bundles or aggregates.

The surfactants and dispersants enhance CNT hydrophilicity, improving their compatibility with the proton exchange membrane and reducing interfacial resistance. This modification facilitates better electron transfer efficiency across the electrode-membrane interface.

### 3.3. Membrane Electrode Assembly Preparation

The MEA was constructed through a multi-step process: proton exchange membrane purification, slurry formulation and homogenization, CNT integration onto porous substrates, thermal stabilization, and final assembly.

*Proton exchange membrane pretreatment.* The Nafion 117 membrane was pretreated to remove surface organic impurities through sequential boiling steps: (1) cut into 2.5 cm × 2.5 cm squares; (2) boil in deionized water for 1 h; (3) boil in $H_{2}O_{2}$ (5 wt%) for one hour; (4) boil in deionized water for one hour, and (5) boil in 0.5 mol/L $H_{2}{\mathrm{SO}}_{4}$ solution for one hour. After each step, the membrane was rinsed 3 to 5 times with deionized water. All boiling procedures were performed at 80 °C in a constant-temperature water bath.

*Conventional microporous layer fabrication.* To establish a baseline and provide a substrate for subsequent CNT modification, a conventional MPL was first fabricated. The MPL slurry was prepared by mixing 288 mg of carbon powder with 0.3 mL of a 10 wt% PTFE solution (to control hydrophobicity) and 3.6 mL of ethylene glycol as the solvent. The mixture was magnetically stirred for 10 min, sonicated for 15 min, and then stirred for an additional 3 h to ensure a homogeneous dispersion. This slurry was uniformly brush-coated onto Toray carbon paper (TGP-H-060), which served as the gas diffusion layer (GDL) substrate for both electrodes. The coated GDLs were then vacuum-dried at 60 °C to remove the solvent, forming a conventional MPL with a carbon loading of approximately 4.7 mg/cm^2^. For the baseline CB-DMFC, this MPL-coated GDL was used directly for catalyst deposition without further modification.

*CNT integration via nitrogen sintering.* An internally porous CNT architecture was engineered using nitrogen-assisted sintering. To achieve optimal and symmetrical performance for both the anodic (methanol oxidation) and cathodic (oxygen reduction) reactions, identical preparation parameters were strictly applied to both electrodes. This included using slurry-coated GDL substrates with precisely controlled and equal precursor loadings for the anode and cathode. These substrates were placed in a tube furnace. The chamber was first evacuated, then purged with high-purity nitrogen at a flow rate of 100–200 sccm to maintain an inert atmosphere. A controlled thermal profile was applied: the temperature was ramped to 800–900 °C at a rate of 5 °C/min. By co-sintering both electrodes within the same thermal batch, uniform structural characteristics (e.g., CNT density, alignment, and layer thickness) were guaranteed. Under these conditions, catalyst nanoparticles decomposed thermally, catalysing the conversion of carbon precursors into structured tubular networks. The furnace was then allowed to cool naturally to preserve structural integrity. This process yielded a symmetrical and conductive CNT carrier matrix with optimized porosity across both electrodes.

**Figure 2:**
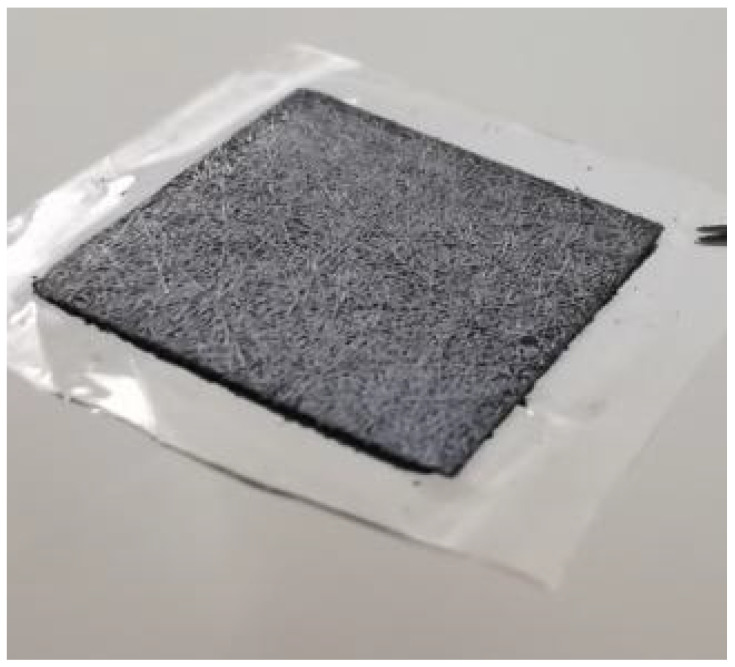
Prepared membrane electrode.

*Electrode assembly.* Catalyst slurries for the electrodes were prepared by blending 60 wt% PtRu/C (anode) or 40 wt% Pt/C (cathode) catalysts with a 5 wt% Nafion solution, isopropanol, and deionized water. To ensure the formation of a homogeneous 3D conductive network, the CNT dispersion within the viscous Nafion solution was achieved by prolonged sonication (24 h). This duration was necessary to overcome the strong van der Waals forces between CNT bundles. A low-power pulsed sonication mode (40 kHz, 150 W) combined with a continuous ice-water bath was employed to prevent structural damage or overheating, maintaining suspension stability for over 48 h and guaranteeing high reproducibility. These catalyst layers were then uniformly deposited onto the CNT-enhanced substrates via ultrasonic spray coating. Residual solvents were removed by thermal consolidation at 313 K for 30 min. The finalized membrane electrode assembly (MEA) was assembled by sandwiching the pretreated Nafion 117 membrane between the anode and cathode. The components were integrated into a cohesive assembly with an active area of 4 cm^2^ by hot-pressing at 135 °C under 1 MPa pressure for five minutes, as shown in Figure 2.

**Figure 3:**
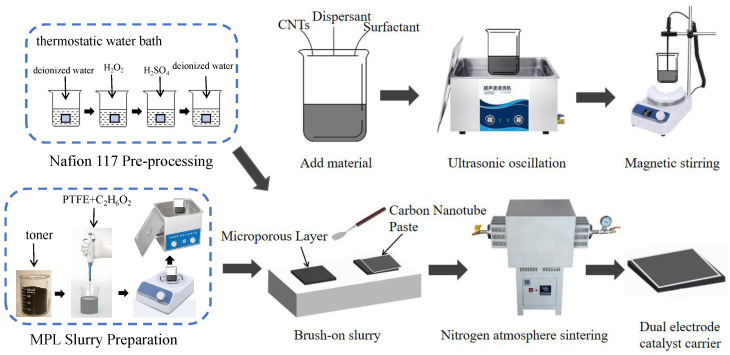
Preparation process of the composite membrane electrode.

The CNT network within the substrates was formed and stabilized through high-temperature nitrogen sintering, as detailed in Section 3.1. This process follows a catalytic carbonization mechanism in which, under an inert N_2_ atmosphere, residual carbon sources rearrange, with pre-deposited Pt-based nanoparticles acting as nucleation sites. This promotes the directional growth of carbonaceous materials into a robust 1D tubular morphology, creating seamless catalyst-support contact. The complete fabrication workflow is illustrated in Figure 3.

**Figure 4:**
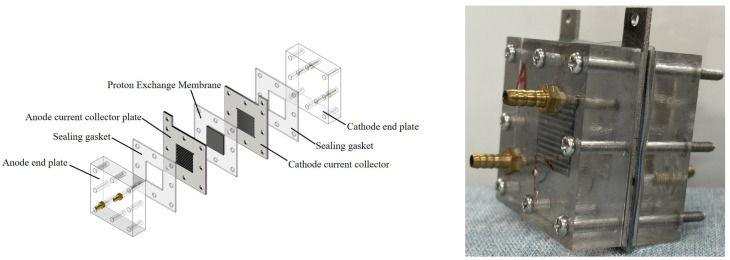
Active DMFC single-cell structure and prepared sample.

*Single-cell integration.* The DMFC single cell employed a MEMS-inspired architecture. It consisted of acrylic end plates (50 mm × 50 mm × 15 mm) featuring serpentine flow channels with a 3 mm diameter. Methanol solution was fed through the anode channels, while the cathode channels managed oxygen ingress and carbon dioxide egress. Current collectors made from 1.5 mm thick 304 stainless steel contained perforated arrays with a 38.4% open area. Thermally resistant silicone gaskets (0.1 mm thick) provided electrical isolation between the MEA and the current collectors. The integrated system is illustrated in Figure 4.

### 3.4. Experimental Setup

**Figure 5:**
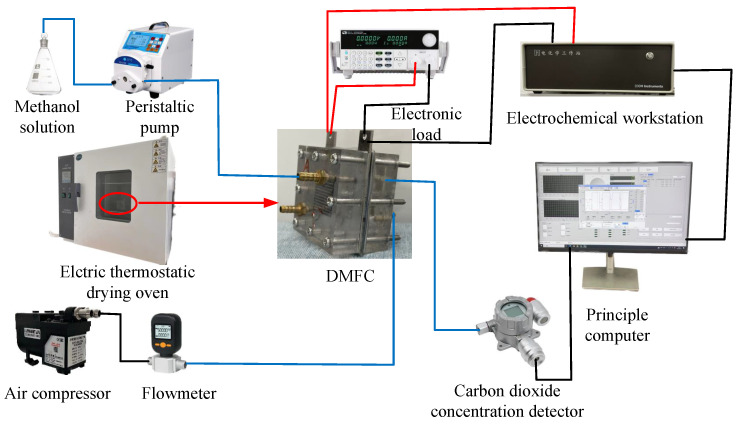
Integrated active DMFC test platform.

The DMFC performance was evaluated using an integrated testing platform comprising six subsystems: (1) a single-cell fixture, (2) a methanol supply unit, (3) an air delivery system, (4) a CO_2_ concentration monitor, (5) a temperature control unit, and (6) an electrochemical measurement station, as shown in Figure 5.

The cell was operated inside a constant-temperature oven maintained at 70 °C to ensure thermal stability. Polarization curves and power densities were obtained using linear sweep voltammetry (LSV) with a programmable DC electronic load, which scanned current densities from 0 to 300 mA/cm^2^ at a rate of 1 mA/s. Electrochemical impedance spectroscopy (EIS) was performed over a frequency range of 100 kHz to 0.1 Hz with a 10 mV amplitude to analyze degradation mechanisms. Methanol crossover was quantified by measuring the CO_2_ concentration in the cathode exhaust using an infrared sensor.

Prior to performance testing, the MEA underwent an in situ activation procedure to optimize proton conductivity and catalyst activity. First, 100 mL of deionized water was slowly injected into the anode side of the assembled DMFC at a flow rate of 3 mL/min using a peristaltic pump to hydrate the membrane. After confirming there were no leaks, the water was drained, and the cell was placed in the 70 °C oven for one hour to equilibrate. The anode was then primed with a 2.5 mol/L methanol solution. The cell was discharged at a constant current density of 50 mA/cm^2^ for one hour, after which the current density was increased by 20 mA/cm^2^ increments every five minutes until the cell voltage fell below the cut-off value.

## 4. Results and Discussion

This section presents and discusses the experimental findings for the CNT-enhanced MEAs. The analysis begins with a detailed microstructural characterization using scanning electron microscopy (SEM), contrasting the conventional MPL morphology with the hierarchical, aligned pore channels of the engineered CNT networks. Subsequently, the electrochemical performance is evaluated through polarization curves and power density measurements across a range of methanol concentrations, elucidating how the CNT-induced porosity governs charge and mass transfer processes. Methanol crossover behaviour is quantified via in situ CO_2_ emission monitoring and correlated with long-term voltage stability over 100-h durability tests. All results are interpreted within the framework of structure-property relationships to establish a mechanistic understanding of the CNT support’s role in enhancing DMFC performance.

### 4.1. Microstructural Characterization

The pore architecture, surface morphology, and catalyst distribution within the GDLs and CLs, features that critically govern mass transfer kinetics and electrochemical activity in DMFCs, were characterized using SEM.

**Figure A1:**
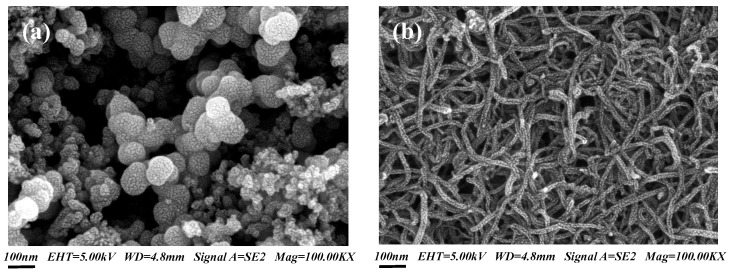
SEM images of the microporous layer: (**a**) MPL at 100,000× magnification; (**b**) CNT-MPL at 100,000× magnification.

*GDL microstructure.* An SEM image of Toray carbon paper coated with a conventional MPL is presented in Figure A1a at 100,000× magnification, where the presence of micropores and particle accumulation patterns is clearly resolved. The irregular surface morphology exhibits high roughness. In contrast, Figure A1b demonstrates CNT-modified MPL sintered in nitrogen atmosphere under identical magnification, exhibiting vertically aligned nanotubes with continuous fibrous architecture. Quantitative analysis confirms three key advantages: (1) higher pore density compared with conventional MPL through interconnected mesochannels, (2) improved dispersion uniformity reducing catalyst particle spacing, and (3) enhanced graphitic crystallinity. This structural hierarchy synergistically enables rapid oxygen diffusion.

**Figure 6:**
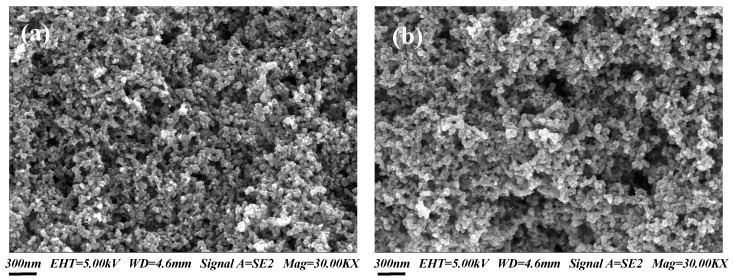
SEM images of the cathode catalyst layer: (**a**) MPL-CCL at 30,000× magnification; (**b**) CNT-CCL at 30,000× magnification.

*Catalyst layer microstructures.* Figure 6a,b compare the cathode catalyst layer microstructures at 30,000× magnification for conventional MPL-supported and CNT-modified architectures, respectively. The conventional CCL exhibits sparse and irregularly shaped pores. In contrast, the CNT-modified CCL displays a uniform distribution of cylindrical pores at a significantly higher density. Granular Nafion ionomer agglomerates show greater surface coverage on the CNT scaffold, indicating enhanced electrolyte permeation through its hierarchical pore network. This expanded CNT scaffold also provides more three-dimensional sites for catalyst attachment, effectively enlarging the triple-phase boundaries. Furthermore, the proton conductivity of Nafion within this structure facilitates deeper catalyst penetration, thereby improving oxygen mass transfer and overall catalyst utilization.

**Figure A2:**
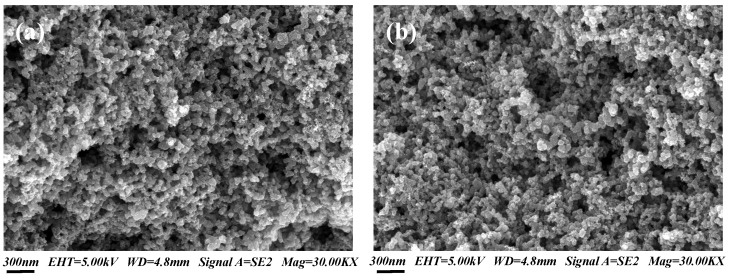
SEM images of the anode catalyst layer: (**a**) MPL-ACL at 30,000× magnification; (**b**) CNT-ACL at 30,000× magnification.

The anode catalyst layer (ACL) exhibits a highly similar microstructure. The CNT-modified ACL shares the uniform, agglomerate-free morphology characteristic of the CNT scaffold (Figure A2 in Appendix B). Compared to the rough and irregular surface of the conventional MPL-ACL, this structurally consistent CNT-supported architecture promotes the formation of smaller, more uniformly distributed catalyst particles. This expands the electrochemically active surface area and enhances the efficiency of the methanol oxidation reaction. Additionally, the smoother and less porous surface of this configuration reduces the effective methanol diffusion coefficient, which helps suppress methanol crossover.

**Figure 7:**
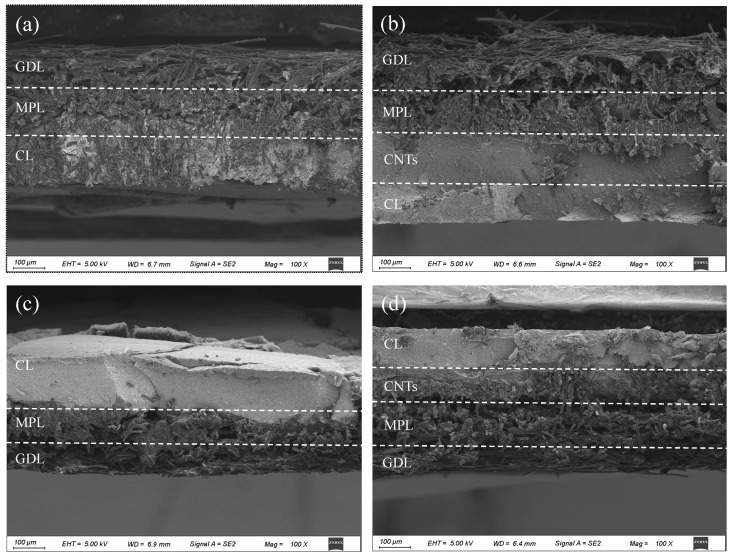
SEM images of cross-sections of two DMFC electrodes: (**a**) CB-DMFC cathode; (**b**) CNT-DMFC cathode; (**c**) CB-DMFC anode; (**d**) CNT-DMFC anode.

*Cross-sectional electrode architecture.* Figure 7 presents cross-sectional SEM images (100× magnification) comparing CB-DMFC and CNT-DMFC electrodes. The CB-DMFC cathode in Figure 7a and anode in Figure 7c exhibit the conventional tri-layer structure: GDL, MPL, and CL. In contrast, the CNT-DMFC electrodes in Figure 7b,d incorporate an additional CNT interlayer between the MPL and the catalyst layer. Note that sample compression during SEM preparation increases the apparent density of the catalyst layers beyond their operational state. Despite this artifact, the CNT-based electrodes appear thicker, likely due to the three-dimensional CNT scaffolding, which provides ample anchoring sites for catalyst particles. This structure promotes uniform PtRu dispersion, suppresses aggregation, and expands the electrochemical active area.

**Figure 8:**
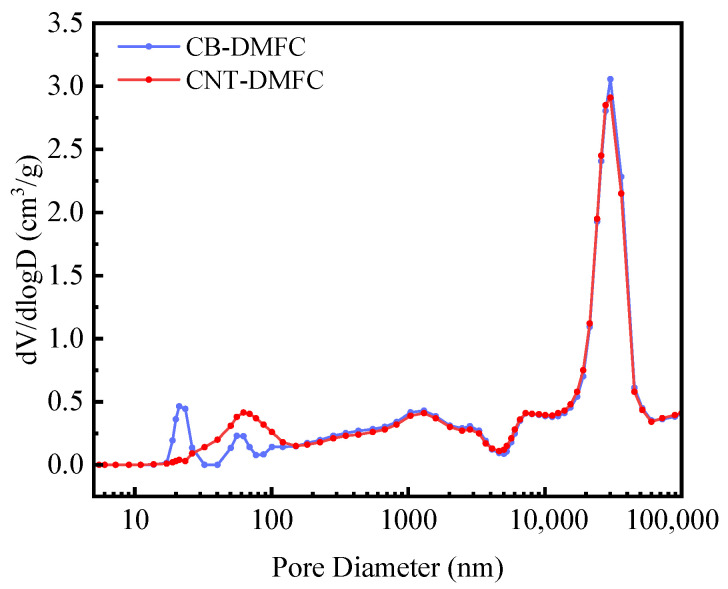
Comparative pore size distributions of the traditional CB and CNT-modified electrodes. The sharp peak at ∼21 nm for the CNT-DMFC signifies an optimized mesoporous network, contrasting with the broad, flooding-prone peak (70–80 nm) of the CB electrode.

*Pore size distribution.* A quantitative comparison of the pore size distributions between the traditional CB electrode and the CNT-modified electrode was conducted to elucidate the underlying structure-performance relationship (Figure 8). Both electrodes share an identical macroporous baseline at approximately 30 μm, originating from the carbon paper substrate. However, a significant divergence occurs in the mesoporous region. The traditional CB electrode exhibits a broad, flat distribution centered around 70–80 nm, characteristic of randomly agglomerated carbon particles and indicative of a structure prone to interfacial flooding. In striking contrast, the CNT-modified electrode eliminates this broad peak, showing instead a highly concentrated and sharp mesoporous peak at approximately 21 nm. This distinct 21 nm pore network, formed by the interwoven CNT architecture, optimizes the capillary pressure gradient within the electrode, thereby facilitating efficient mass transport. This comparative structural analysis provides a direct physical explanation for the substantially reduced mass transfer resistance ($R_{4}$) and the superior power density observed in the CNT-DMFC system.

### 4.2. Cyclic Voltammetry Analysis

**Figure 9:**
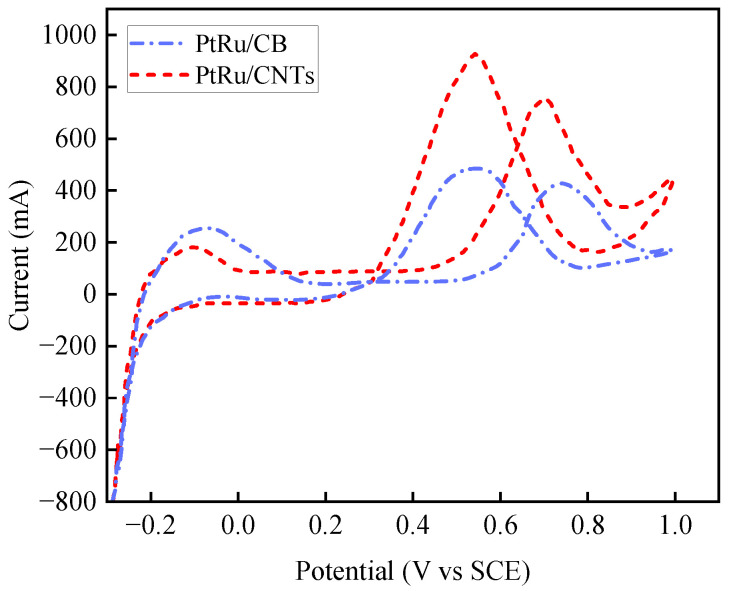
CV curves recorded in 0.5 mol/L H_2_SO_4_ at a scan rate of 50 mV/s (−0.3 to 1.0 V vs. SCE).

CV was employed to calculate the ECSA, quantifying catalyst utilization in DMFCs before and after modification. Experiments were conducted in a 0.5 mol/L H_2_SO_4_ electrolyte using a standard three-electrode setup (working electrode, saturated calomel reference electrode (SCE), and auxiliary electrode). Prior to testing, dissolved oxygen was removed by purging the electrolyte with high-purity N_2_ for at least 30 min. CV scans were performed over a potential range of −0.3 to 1.0 V (vs. SCE) at a scan rate of 50 mV/s for 30–50 cycles. Initial cycles activated the electrode surface until stable curves were obtained. Figure 9 presents representative current–potential curves for the PtRu/CB and PtRu/CNTs catalysts.

Analysis of the CV curves reveals distinct electrochemical behaviors. In the hydrogen adsorption/desorption region (−0.3 to 0.2 V), the PtRu/CNTs catalyst exhibited sharper, more intense current peaks, indicating a greater number of exposed active sites due to improved dispersion on the high-surface-area CNT support. Conversely, PtRu/CB showed broader peaks with lower current magnitudes, suggesting catalyst agglomeration and reduced accessible surface area. In the methanol oxidation region (0.2 to 1.0 V), both catalysts exhibited characteristic oxidation peaks near 0.5 V and 0.65 V, corresponding to the oxidation of adsorbed CO-like intermediates. The key difference is in the peak current density: the main oxidation peak for PtRu/CNTs reached about 0.95 A, substantially higher than the 0.5 A for PtRu/CB, confirming superior intrinsic catalytic activity on the CNT support.

The ECSA was quantified from the charge associated with hydrogen underpotential deposition. The faradaic current in the hydrogen adsorption region (−0.3 to 0.2 V) was integrated to obtain the hydrogen adsorption charge ($Q_{H}$). Values of $Q_{H}$ = 15 mC for PtRu/CNTs and 8 mC for PtRu/CB were obtained. These values were substituted into Equation (1), using a standard charge density of $q_{H}$ = 210 μC/cm^2^ for a monolayer of hydrogen adsorbed on polycrystalline Pt. The calculated ECSA values are 35.7 m^2^/g for PtRu/CNTs and 19.0 m^2^/g for PtRu/CB. This represents an approximately 88% increase in active surface area, unequivocally demonstrating that the CNT support enhances catalyst utilization. The enhancement in ECSA serves as a robust quantitative indicator of improved catalyst dispersion and reduced particle agglomeration. While direct nanoscale imaging (e.g., HRTEM) was not performed, the result aligns with the established literature [31,32]. Studies have demonstrated that the unique surface chemistry, high specific area, and curvature of CNTs effectively confine Pt-based nanoparticles, typically within the 2–4 nm range. This nanoscale confinement mitigates Ostwald ripening and agglomeration, which are common on conventional carbon black supports, directly corroborating the observed increase in active site availability and contributing to the enhanced dynamic discharge stability of the CNT-DMFC system.

### 4.3. Output Power Evaluation

The electrochemical output performance of conventional CB-DMFC and CNT-DMFC was evaluated by recording polarization curves via LSV under quasi-steady-state conditions. During testing, cells were maintained at 70 °C. The current density was increased from open-circuit in 5 mA/cm^2^ increments, with the voltage stabilized for 20 s at each step before data acquisition. To ensure data reliability, each MEA was activated for 12 h prior to testing. Measurement uncertainty was controlled by instrumental precision: the calibrated electronic load system provided an accuracy within ±1 mV for voltage and ±1 mA for current. During polarization sweeps, each data point was recorded after maintaining the current density for 3 min to ensure stable voltage output.

**Figure 10:**
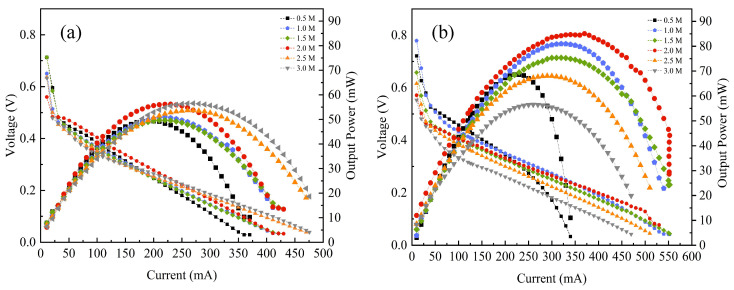
Polarization and power-density curves of DMFCs at different methanol concentrations: (**a**) CB-DMFC; (**b**) CNT-DMFC.

Figure 10 compares the polarization and power density curves for both configurations across methanol concentrations from 0.5 to 3.0 mol/L. Under these conditions, both systems exhibited peak power at an optimal concentration of 2.0 mol/L, with performance declining at higher concentrations due to exacerbated methanol crossover. At this optimum, the CNT-DMFC achieved a maximum power density of 85.12 mW/cm^2^, representing a 51.1% enhancement over the 56.35 mW/cm^2^ delivered by the CB-DMFC. This improvement is attributed to the CNT support structure, which enhances catalyst utilization, mass transport, and charge transfer kinetics.

*Low-current-density region.* In the low-current-density regime (<50 mA/cm^2^), performance is governed by activation polarization. The CNT-DMFC shows a steeper initial slope in the polarization curve than the CB-DMFC, as depicted in Figure 10b, attributed to interfacial phenomena within the CNT/ionomer network. The hierarchical CNT network creates tortuous diffusion pathways, which locally restrict methanol concentration at the catalyst surface. This structure simultaneously suppresses methanol crossover. To quantify this effect, in situ CO_2_ emissions at the cathode exhaust, a direct proxy for permeated methanol, were monitored using an infrared sensor. As summarized in Table 2, at 2.0 mol/L, the CNT-DMFC exhibited CO_2_ emissions of 2551–3600 ppm, a significantly reduced range compared to 1482–5673 ppm for the CB-DMFC. This confirms the CNT support effectively mitigates crossover, supporting our mass transport model and explaining the enhanced cell efficiency.

*Mid-current-density region.* Within the medium current density range (50 to 150 mA/cm^2^), ohmic losses dominate. Despite the high axial conductivity of individual CNTs, the CNT-DMFC exhibits a more pronounced ohmic voltage drop. This is likely due to increased interfacial contact resistance within the CNT network and between CNTs and catalyst particles, as well as to potential surface defects introduced during synthesis. These imperfections reduce effective electron-conduction pathways, increasing total impedance by 28% compared to CB-DMFC (see EIS data in Section 4.4), which limits power output despite the intrinsic conductivity of CNTs.

### 4.4. Impedance Characteristics

**Figure 11:**
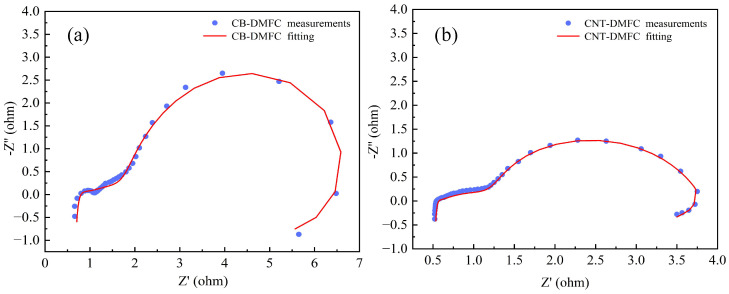
Nyquist plots from EIS: (**a**) CB-DMFC; (**b**) CNT-DMFC.

EIS was employed to quantify the impact of the CNT-enhanced MEA structure on the internal charge and mass transport resistances within the DMFC. Measurements were conducted at the cell operating temperature of 70 °C under a constant discharge current density of 1.25 mA/cm^2^, with an alternating perturbation frequency range from 0.01 Hz to 100 kHz. A four-terminal configuration was used to minimize measurement artifacts: the cathode was connected to the working and sense electrodes, while the anode was connected to the counter and reference electrodes. As shown in the Nyquist plots (Figure 11), the CNT-DMFC exhibits a smaller total impedance semicircle compared to the conventional CB-DMFC, indicating superior overall charge transfer efficiency and reduced polarization losses.

**Figure 12:**
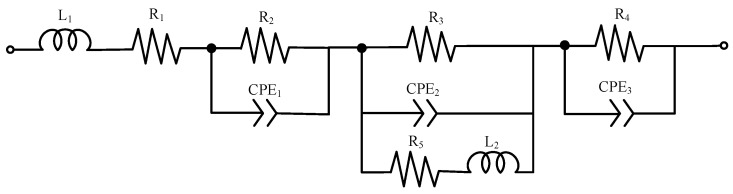
Equivalent circuit model used for fitting the electrochemical impedance data.

To deconvolute the contributions of distinct physical and electrochemical processes to the total impedance, the EIS data were fitted using an ECM based on established models [33,34], and depicted in Figure 12. To accurately capture the non-ideal capacitive behavior and surface heterogeneity of the highly porous CNT-based electrodes, constant-phase elements (CPEs) were used instead of ideal capacitors. The ECM components and their rigorously defined physical significance are as follows:

- $L_{1}$: High-frequency inductance from measurement cables and external connections.
- $R_{1}$: Ohmic resistance representing contact impedance at the bipolar plate/GDL interface.
- $R_{2}$: Contact resistance at the membrane/catalyst layer interface.
- $R_{3}$: Charge transfer resistance for the methanol oxidation (anode) and oxygen reduction (cathode) reactions.
- $R_{4}$: Mass transfer resistance associated with the diffusion of methanol/protons and product removal.
- $R_{5}$: Resistance attributed to intermediate species adsorption and reaction byproducts (e.g., CO).
- $L_{2}$: Low-frequency inductance, often linked to reaction intermediates or impurities.
- ${\mathrm{CPE}}_{1}$: Pseudocapacitance of the membrane-catalyst layer composite.
- ${\mathrm{CPE}}_{2}$ and ${\mathrm{CPE}}_{3}$: Distributed double-layer capacitance at the anode and cathode catalyst-electrolyte interfaces, respectively.

By systematically assigning these physical origins to each circuit element, this multi-component model provides a robust mathematical framework. It ensures that the extracted resistance values accurately reflect specific internal processes rather than mathematical artifacts, thereby justifying its application in evaluating DMFC performance and degradation.

Non-linear least-squares fitting was performed on the EIS data. The fitted parameters for both DMFC configurations are summarized in Table 3, revealing distinct differences in the contributions of individual resistive elements.

The fitted parameters reveal two key increases in resistance for the CNT-DMFC. First, the 78.6% rise in contact resistance ($R_{1}$) stems from the structural complexity of the CNT interlayer. As shown in cross-sectional SEM (Figure 9), a thicker catalyst layer with additional CNT-matrix interfaces elongates the electron-conduction pathway from the current collector, increasing the number of interfacial junctions and thus the overall contact resistance, despite the high intrinsic conductivity of CNTs. It is important to note that identical hot-pressing parameters (135 °C, 1 MPa, 5 min) were applied to both MEAs. For the inherently thicker CNT-based electrodes, this standard pressure and duration may be insufficient for optimal interfacial consolidation. We postulate that $R_{1}$ could be mitigated in future work by tailoring hot-pressing conditions (e.g., higher pressure or longer duration) specifically for the CNT architecture. The 49.9% increase in reaction byproduct resistance ($R_{5}$) is more nuanced. The extended, tortuous diffusion paths within the CNT network, while beneficial for suppressing methanol crossover, also reduce the local methanol concentration at the catalyst surface (per Fick’s law). This altered local environment can increase the coverage or stability of adsorbed intermediate species like CO, thereby elevating the associated resistance.

*Analysis of decreased resistances.* Conversely, the CNT support structure delivers substantial reductions in three critical resistances. The 22.2% decrease in $R_{2}$ (membrane-catalyst interface) stems from the uniform catalyst dispersion and improved interfacial bonding promoted by the CNT scaffold. The 27.0% reduction in charge-transfer resistance $R_{3}$ is a direct result of the increased ECSA and enhanced electrical connectivity, which facilitate electron transfer during the redox reactions. Most significantly, the 53.7% drop in mass transfer resistance $R_{4}$ is achieved through the hierarchical pore structure of the CNT layer, which promotes efficient water removal via capillary action, mitigates flooding, and improves the transport of reactants and products.

*Overall impedance and system trade-off.* The net effect of these competing changes is a 5.2% reduction in the total fitted resistance, from 12.069 Ω for the CB-DMFC to 11.437 Ω for the CNT-DMFC. This result underscores a critical design trade-off. The architecture willingly accepts increases in certain interfacial ($R_{1}$) and local reaction ($R_{5}$) resistances to achieve far greater reductions in the resistances that fundamentally limit performance: charge transfer ($R_{3}$) and, most importantly, mass transfer ($R_{4}$). This strategic compromise is pivotal for enhancing overall mass transport kinetics, suppressing electrode flooding, and delivering the observed gains in power density and operational stability.

### 4.5. Discharge Performance and Durability Assessment

The long-term discharge performance and durability of the DMFCs were evaluated using an accelerated aging protocol designed to simulate real-world dynamic operation, specifically referencing the China light-duty vehicle test cycle (CLTC). By applying dynamically controlled load currents that replicate driving profiles, the service life and performance degradation of both CB-DMFC and CNT-DMFC configurations were assessed. The CLTC profile consists of low-, medium-, and high-speed segments, each comprising four operational states: acceleration, constant speed, deceleration, and idle [35]. To accurately capture the stochastic nature of real driving, a Markov process was used to randomize the sequence of these states according to their CLTC occurrence probabilities, thereby generating realistic transient load dynamics.

Due to the power limitations of a single fuel cell, the absolute load currents were scaled down proportionally while preserving the relative duty cycles of each CLTC state. The nine distinct loading currents corresponding to the driving states (excluding idle) are detailed in Table 4. For instance, currents ranged from 0.018 A for low-speed deceleration to 0.138 A for high-speed acceleration.

**Figure 13:**
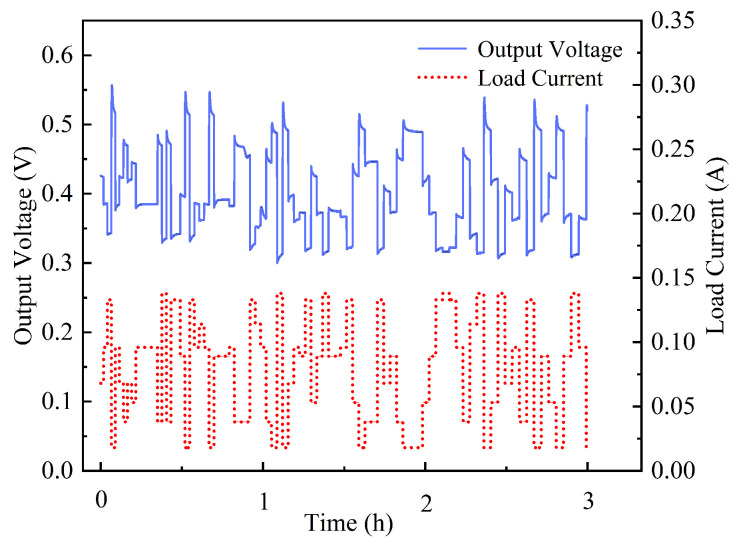
DMFC voltage response under a CLTC-simulated dynamic load profile.

Figure 13 illustrates a representative three-hour voltage response under the CLTC-simulated load profile. The dashed line denotes the randomly sequenced load current generated by the Markov process, while the solid line tracks the corresponding DMFC output voltage. In an active fuel cell system with peristaltic-pump-driven methanol supply, the output voltage remained stable with fluctuations within ±2.1%, demonstrating the system’s robustness under dynamic operating conditions.

Long-term durability tests were conducted at 70 °C with a 2.0 mol/L methanol solution, applying the randomized CLTC load profile. Performance degradation was monitored via the decay in output power, with end-of-life defined as the point at which power dropped below 10% of its initial value (in order to make full use of the available testing data). Tests were conducted in daily five-hour cycles until failure.

**Figure 14:**
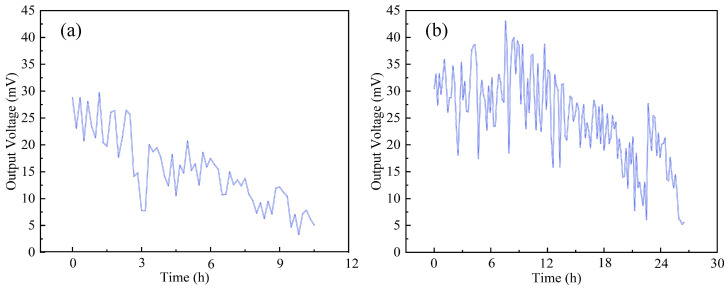
Long-term power output decline trends: (**a**) CB-DMFC; (**b**) CNT-DMFC under CLTC-based dynamic loading.

Figure 14 contrasts the degradation trajectories. The CB-DMFC exhibited rapid decay with an average voltage loss rate of approximately 48 μV/h. Post-test analysis attributed this primarily to Nafion ionomer loss, which reduced the triple-phase boundary length and decreased the ECSA by about 37%. In contrast, the CNT-DMFC degraded more gradually (28 μV/h). This improvement benefits from the structural stability and high electronic conductivity of the CNT support, which helps maintain catalyst activity and interface integrity. However, the observed degradation indicates that other intrinsic mechanisms, such as Nafion ionomer degradation and catalyst coarsening, still limit ultimate long-term performance and commercialization potential.

**Figure 15:**
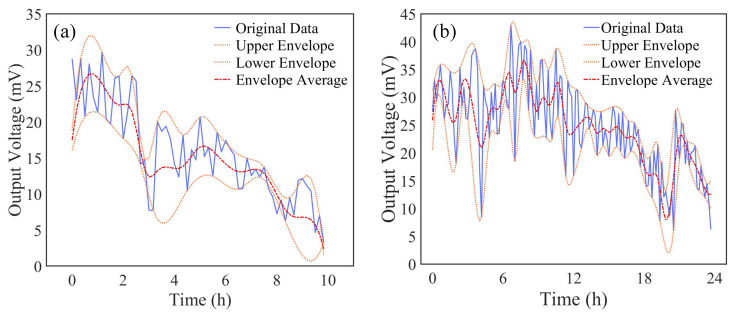
Envelope-averaged output power trends for long-term degradation analysis: (**a**) CB-DMFC; (**b**) CNT-DMFC.

To quantify the degradation kinetics, the power data were processed to obtain envelope-averaged trends, as shown in Figure 15. The CNT-DMFC exhibited a 40% slower average power decay rate (0.32%/h) compared to the CB-DMFC (0.54%/h), confirming the superior structural resilience of the CNT-based electrode despite potential long-term graphitization loss. This performance enhancement aligns with the impedance-based trade-off identified earlier: the architecture tolerates increases in certain resistances (e.g., $R_{5}$, associated with intermediate species) to achieve greater reductions in mass transfer resistance ($R_{4}$). This strategic compromise ultimately extended the operational service life of the CNT-DMFC by approximately 67% compared to the conventional counterpart.

## 5. Conclusions

This work developed a novel dual-electrode catalyst layer architecture based on carbon nanotubes to overcome the low catalyst utilization and poor durability limitations of conventional direct methanol fuel cells. The key findings are summarized as follows:

- The CNT-DMFC achieved a 51.1% higher peak power density than the conventional CB-DMFC under dynamic operating conditions simulated by the CLTC cycle. The engineered electrode architecture effectively mitigates electrode flooding, leading to an extended discharge duration and enhanced operational stability.
- The CNT support structure promotes uniform PtRu dispersion and facilitates reactant/product diffusion. This synergistic effect substantially reduces critical resistances: membrane-catalyst interface resistance by 22.2%, charge-transfer resistance by 27.0%, and mass-transfer resistance by 53.7%, improving overall reaction kinetics.
- The CNT-based microporous layer creates a hierarchical pore architecture that expands the triple-phase boundaries. While this structural complexity increases contact resistance by 78.6% and intermediate species resistance by 49.9%, the net effect is a decisive enhancement in multiphase transport capability.

## Figures and Tables

**Table 1:** Comparative electrocatalytic performance of carbon-based supports.

Electrode Material	Current Density	Peak Potential	ECSA	Ref.
Composition	(mA cm^−2^)	(V)	(m^2^ g^−1^ Pt)	
Pt/C-OT (Ozone Treated Carbon) at 60 °C	9.070	0.650	64.41	[10]
Pt/C-OT at 100 °C	13.210	0.450	70.25	[10]
Pt/C-OT at 140 °C	17.780	0.450	78.66	[10]
Au/C	0.500	−	−	[11]
PtPd NCs/C	1.770	0.494	67.00	[12]
PtPd NAs/C	0.608	0.450	69.00	[12]
PtRu/C	0.530	0.378	61.00	[12]
Pt/C	0.530	0.370	63.00	[12]
PtRu/C	0.079	0.613	3.25	[13]
PtRu/TiO_2_-GA	0.554	0.676	23.43	[13]
PtRu/TiO_2_-GAOPt	0.568	0.693	30.63	[13]
PtCo/NH_2_-rGO	0.086	0.245	−	[14]

**Table 2:** In situ CO_2_ emissions at the cathode exhaust for CB-DMFC and CNT-DMFC.

Methanol Concentration (mol/L)	CB-DMFC (ppm)	CNT-DMFC (ppm)
0.5	1080–2260	767–1960
1.0	1814–2870	1280–1323
1.5	2731–3335	2063–2351
2.0	1482–5673	2551–3600
2.5	3397–4500	2063–2104
3.0	4207–5785	3335–4137

**Table 3:** Fitted parameters for the equivalent circuit model extracted from EIS data.

Parameter	CB-DMFC (Ω)	CNT-DMFC (Ω)	Change
$R_{1}$ (Contact)	0.07	0.125	+78.6%
$R_{2}$ (Membrane catalyst)	0.787	0.612	−22.2%
$R_{3}$ (Charge transfer)	6.714	4.9	−27.0%
$R_{4}$ (Mass transfer)	1.297	0.6	−53.7%
$R_{5}$ (Reaction byproduct)	3.469	5.2	+49.9%

**Table 4:** CLTC-based loading current settings for dynamic durability testing.

Speed Segment	Operating State	Duty (%)	Avg. Speed (km/h)	Loading (A)
Low	Acceleration	26.27	35.02	0.053
	Even speed	24.31	24.91	0.038
	Deceleration	20.65	11.85	0.018
	Idling	28.77	0	0
Medium	Acceleration	29.95	62.49	0.096
	Even speed	29.38	55.53	0.089
	Deceleration	22.39	44.52	0.068
	Idling	18.28	0	0
High	Acceleration	17.47	90.92	0.138
	Even speed	49.92	87.71	0.133
	Deceleration	27.44	75.13	0.114
	Idling	5.17	0	0

## Data Availability

The original contributions presented in this study are included in the article. Further inquiries can be directed to the authors.

## Abbreviations

The following abbreviations are used in this manuscript:

ACL	Anode catalytic layer
CB	Carbon black
CCL	Cathode catalytic layer
CL	Catalytic layer
CLTC	China light-duty vehicle test cycle
CNF	Carbon nanofibre
CNT	Carbon nanotube
CPE	Constant-phase element
CV	Cyclic voltammetry
DMFC	Direct methanol fuel cell
ECSA	Electrochemical active surface area
EIS	Electrochemical impedance spectroscopy
GA	Graphene aerogel
GAOPt	Graphene aerogel optimized Pt
GDL	Gas diffusion layer
LSV	Linear scanning voltammetry
MEA	Membrane electrode assembly
MOR	Methanol oxidation reaction
MPL	Micro porous layer
NAs	Nanoassemblies
NCs	Nanocubes
NMP	N-methyl-pyrrolidone
ORR	Oxygen reduction reaction
OT	Ozone-treated
PTFE	Polytetrafluoroethylene
PVA	Polyvinyl alcohol
rGO	Reduced graphene oxide
SCE	Saturated calomel electrode
SEM	Scanning electron microscopy

## Appendix A. Reaction Kinetics and Mass Transfer Limitations

During electrochemical reactions, the current density is governed by reaction kinetics and mass transfer limitations. The Butler–Volmer equation describes the relationship between overpotential and current density [36]:

(A1) $$j=j_{0}\left[\exp\left(\frac{\alpha nF}{RT}\eta \right)-\exp\left(-\frac{(1-\alpha )nF}{RT}\eta \right)\right]$$

where $j_{0}$ is the exchange current density; α is the transfer coefficient; n=6 is the electron transfer count for methanol oxidation; η is the overpotential; *F* is Faraday’s constant; *R* is the gas constant; and *T* is the temperature. According to Faraday’s laws of electrolysis, the current directly measures the reaction rate:

(A2) $$i=nF\frac{dN}{dt}$$

where *i* is the current and dN/dt is the substance consumption or production rate. The total current relates to the active reaction area is as follows:

(A3) $$i=j\cdot A_{\mathrm{Pt}}$$

where *j* is the current density per active site, and $A_{\mathrm{Pt}}$ is the total electrochemically active surface area of the catalyst.

Methanol transport through porous electrodes follows Darcy’s law for convective flow:

(A4) $$Q=KA\frac{h_{2}-h_{1}}{L}$$

where *Q* is the volumetric flow rate; *K* is the hydraulic permeability; *A* is the cross-sectional area; $h_{2}-h_{1}$ is the hydraulic head difference; and *L* is the flow path length. Concurrently, methanol diffusion is governed by Fick’s first law:

(A5) $$J=-D\frac{\partial C}{\partial x}$$

where *J* is the diffusion flux; *D* is the effective diffusion coefficient; and ∂C/∂x is the methanol concentration gradient. The relative dominance of each mechanism depends on operating conditions such as flow rate and concentration.

The conductivity of the CNT network follows percolation theory [37]:

(A6) $$A\propto \frac{{\left(\phi -\phi_{c}\right)}^{t}}{\alpha \cdot R_{2}}$$

where ϕ is the CNT volume fraction; $\phi_{c}$ is the percolation threshold; *t* is a critical exponent; α is an orientation factor; and $R_{2}$ is the inter-CNT contact resistance.

Electrochemical impedance spectroscopy (EIS) models degradation mechanisms [26]:

(A7) $$Z=\frac{V_{0}\sin(\omega t)}{I_{0}\sin(\omega t+\varphi )}$$

where $V_{0}$ and $I_{0}$ are the voltage and current amplitudes, ω is the angular frequency, and φ is the phase shift. The total internal resistance is as follows:

(A8) $$R_{\mathrm{int}}=\sum_{i=1}^{5}R_{i}$$

where $R_{1}$ is the ohmic resistance; $R_{2}$ is the contact resistance; $R_{3}$ is the byproduct-induced resistance; $R_{4}$ is the mass transfer resistance; and $R_{5}$ is the charge transfer resistance. Note that $R_{3}$ and $R_{4}$ may couple through pore blockage effects.

The high specific surface area of CNTs enables superior catalyst dispersion and active-site exposure, thereby reducing the charge-transfer resistance $R_{5}$. Their enhanced electrical conductivity accelerates electron transfer, increasing the current density *j* and reducing the activation overpotential η. These effects collectively elevate the voltage *U* and power output *P* in DMFCs.
